# Zinc oxide nanoparticles harness autophagy to induce cell death in lung epithelial cells

**DOI:** 10.1038/cddis.2017.337

**Published:** 2017-07-27

**Authors:** Jun Zhang, Xia Qin, Bin Wang, Ge Xu, Zhexue Qin, Jian Wang, Lanxiang Wu, Xiangwu Ju, Diptiman D Bose, Feng Qiu, Honghao Zhou, Zhen Zou

**Affiliations:** 1 Institute of Life Sciences, Chongqing Medical University, Chongqing 400016, China; 2 Department of Pharmacy, The First Affiliated Hospital of Chongqing Medical University, Chongqing 400016, China; 3 Department of Cardiology, XinQiao Hospital, Third Military Medical University, Chongqing 400037, China; 4 National Center for Science and Technology Evaluation (NCSTE), Beijing 100081, China; 5 State Key Laboratory of Medical Molecular Biology, Institute of Basic Medical Sciences, Chinese Academy of Medical Sciences, and Department of Biochemistry and Molecular Biology, Peking Union Medical College, Tsinghua University, Beijing 100005, China; 6 Department of Pharmaceutical and Administrative Sciences, College of Pharmacy, Western New England University, Springfield, MA 01119, USA

## Abstract

Although zinc oxide nanoparticles (ZnONPs) are widely used, they have raised concerns of toxicity in humans. Previous studies have indicated that reactive oxygen species (ROS) and autophagy are involved in the cytotoxicity of ZnONPs, but the regulatory mechanisms between autophagy and ROS remain to be elucidated. Herein, we comprehensively investigated the regulatory mechanism of autophagy and the link between autophagy and ROS in ZnONPs-treated lung epithelial cells. We demonstrated that ZnONPs could induce autophagy, and this process could enhance the dissolution of ZnONPs in lysosomes to release zinc ions. Sequentially, zinc ions released from ZnONPs were able to damage not only lysosomes, leading to impaired autophagic flux, but also mitochondria. Impaired autophagic flux resulted in the accumulation of damaged mitochondria, which could generate excessive ROS to cause cell death. We further demonstrated that the inhibition of autophagy by either pharmacological inhibitors or small interfering RNA (siRNA)-mediated knockdown of Beclin-1 and AMP-activated protein kinase could ameliorate ZnONPs-induced cell death. Moreover, we found that lysosomal-associated membrane protein 1/2 (LAMP-1/2), which were the most abundant highly glycosylated protein in late endosomes/lysosomes, exhibited aberrant expression pattern upon treatment with ZnONPs. Intriguingly, LAMP-2 knockdown, but not LAMP-1 knockdown, could exacerbate the ROS generation and cell death induced by ZnONPs treatment. Meanwhile, LAMP-2 overexpression alleviated ZnONPs-induced cell death, suggesting that LAMP-2 was linked to this toxic phenotype induced by ZnONPs. Our results indicate that autophagic dysfunction could contribute to excessive ROS generation upon treatment with ZnONPs in lung epithelial cells, suggesting that modulating the autophagy process would minimize ZnONPs-associated toxicity.

Nanotechnology has made remarkable progress in recent years, and the global nanotechnology market is estimated to reach a trillion dollars annually by 2015.^1^ Zinc oxide nanoparticles (ZnONPs) are one of the most important metal oxide nanoparticles and its worldwide production is estimated to be up to 1 million tons per year.^2^ They are extensively used not only for industrial/household applications (cosmetics, pigments, coatings, electronic devices, catalysts)^3^ but also for clinical purposes.^4^ However, because of the large-scale production and increasing utilization, concerns of ZnONPs toxicity is raising.^5, 6, 7^ Airway exposure is the general exposure route besides dermal exposure. The respiratory system is vulnerable as it directly and constantly contacts with the environment;^8^ it has been reported that ZnONPs inhalation would cause metal fume fever in humans.^9^

Previous studies have revealed several possible reasons for ZnONPs-related toxicity. Among these, ROS and inflammation caused by ZnONPs are most commonly accepted.^8, 10, 11, 12^ Another well-known toxicity-related mechanism is the release of zinc ions from ZnONPs, which can induce organelle damage in the biological environment.^13, 14, 15^ However, the opinions about whether apoptotic cell death is induced by ZnONPs treatment are controversial. This disparity might be due to the differences in ZnONPs characteristics, doses or cells.^16, 17, 18, 19, 20^

In recent times, autophagy has been considered as a possible mechanism underlying nanomaterial-induced toxicity.^21^ Autophagy (specifically macroautophagy) is thought to begin as a phagophore, followed by interaction with Atg5–Atg12 conjugation. Then, LC3 inserts into the extending phagophore membrane to facilitate the engulfment of intracellular cargos (including aberrant organelles, proteins, virus and nanomaterials) in the double-membrane structure referred to as autophagosomes. The autophagosomes may further fuse with lysosomes to form autolysosomes, and the intracellular contents will be degraded in the lysosomes and recycled.^22, 23, 24^ The integrity of lysosomes is crucial for autophagy. Lysosomal acid environment and numerous hydrolases inside lysosomes facilitate intracellular content degradation. The dynamic process of autophagosomes formation, fusion of autophagosomes with lysosomes and intracellular content degradation, is referred to as autophagic flux, which reflects the rate of autophagic degradation. Lysosomal dysfunction can lead to incomplete autophagy.^25^

It is reported that ZnONPs might induce autophagy,^16, 26^ or autophagic cell death,^27^ but whether ZnONPs indeed induce autophagy is not addressed. On the other hand, Cho *et al.*^13^ have demonstrated that ZnONPs are dissolved more rapidly in an acidic environment, suggesting that ZnONPs may be dissolved in lysosomes and cause lysosomal destabilization in differentiated THP-1 cells. Mihai *et al.*^14^ have demonstrated that zinc ions accumulates specifically in endosomes and lysosomes of alveolar epithelial cells upon ZnONPs treatment.^14^ These results show that ZnONPs can be dissolved in lysosomes and cause lysosomal dysfunction. However, the regulatory mechanism and biological effect of lysosomal dysfunction remain poorly characterized.

Components involved in autophagy are sophisticatedly orchestrated at the molecular level; thus, autophagy can be regulated at multiple steps. The LC3II/LC3I ratio increase or autophagosomes accumulation may result from either autophagy induction or impairment of autophagic flux.^28^ Hence, integrated assays should be performed to clarify whether autophagy is induced by ZnONPs. Furthermore, the subsequent effects from autophagic flux blockade should be evaluated cautiously. Mitophagy, autophagy-dependent degradation of the mitochondria, is important for maintaining the integrity of mitochondria and the balance of ROS.^29^ When autophagic flux is blocked, the elimination of damaged mitochondria will also be impaired, which might be the nexus of autophagy and ROS.

In the present study, we demonstrated that ZnONPs could be delivered into lysosomes via the autophagy pathway and subsequently dissolved in lysosomes to release zinc ions, which was the crucial factor responsible for the ZnONPs-induced cytotoxicity. Moreover, zinc ions could damage mitochondria and lysosomes, further disrupting the negative feedback mechanisms between ROS and mitophagy, leading to damaged mitochondria accumulation, excessive ROS production and cell death.

## Results

### Release of zinc ion from ZnONPs induces cell death in respiratory epithelial cells

The characteristics of ZnONPs used in this study were examined by transmission electron microscopy (TEM) (Supplementary Figure S1a), and the results showed that the particle size of ZnONPs was ~50 nm and the morphology was roughly spherical. These nanoparticles have been well characterized previously. Roy *et al.*^30^ reported that the zeta potential of this ZnONPs was −11.5 mV, with dynamic light scattering (DLS) size distribution of an average diameter of 278.8 nm, in culture media supplemented with 10% serum.^30^

**Figure 1:**
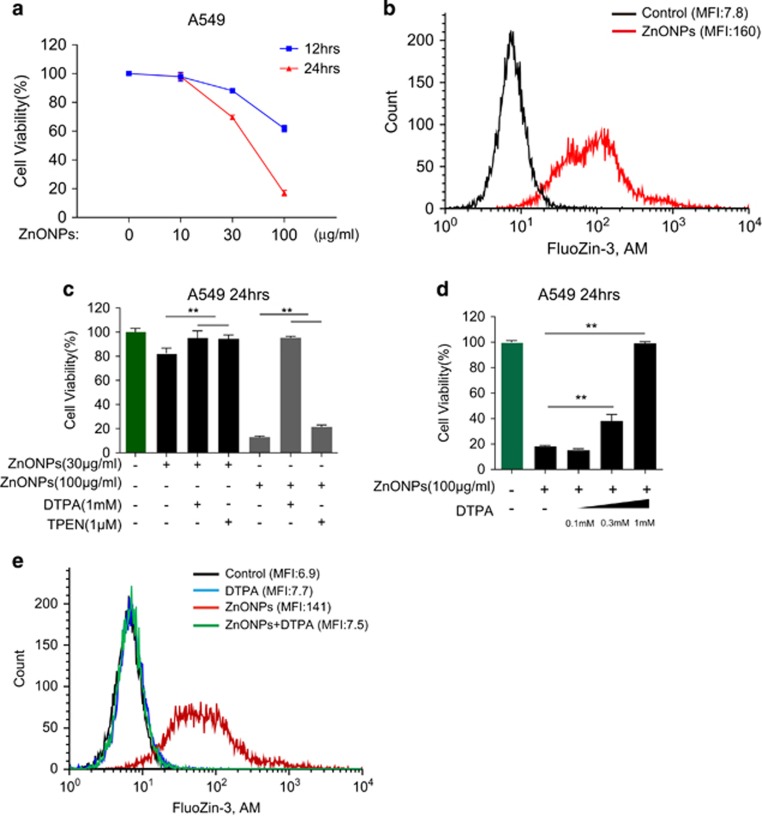
Zinc ion is the key mediator of ZnONPs-induced cytotoxicity. (**a**) MTS analysis of A549 cells treated with ZnONPs at doses of 10, 30 and 100 *μ*g/ml. Cell viability was detected at 12 and 24 h after ZnONPs treatment. Data are representative of three independent experiments (*n*=6 for each group) and values are expressed in mean±S.E.M. (**b**) FACS analysis of cells stained with FluoZin-3, AM. The effect of 30 *μ*g/ml ZnONPs treatment on intracellular zinc ions concentration was analyzed 24 h after treatment. Data are representative of three independent experiments. MFI=mean fluorescence intensity. (**c**) A549 cells were pretreated with 1 mM DTPA or 1 *μ*M TPEN, followed by 30 or 100 *μ*g/ml ZnONPs treatment. MTS analysis of A549 cells was performed at 24 h after treatment. Data are representative of three independent experiments (*n*=6 for each group) and values are expressed in mean±S.E.M. **P<0.01. (**d**) A549 cells were pretreated with 0, 0.1, 0.3 and 1 mM DTPA, followed by 100 *μ*g/ml ZnONPs treatment. MTS analysis of A549 cells was then performed at 24 h after treatment. Data are representative of three independent experiments (*n*=6 for each group) and values are expressed as mean±S.E.M. **P<0.01. (**e**) A549 cells were pretreated with vehicle or 1 mM DTPA, followed by 30 *μ*g/ml ZnONPs treatment. FACS analysis of cells stained with FluoZin-3, AM was performed at 24 h after treatment. Data are representative of three independent experiments

Respiratory epithelial cells are recognized as the first barrier and vulnerable when exposed to high concentrations of nanoparticles. Because damage of alveolar epithelial cells is the hallmark event of acute lung injury,^31^ we chose the human lung adenocarcinoma cell line A549, which is the classical and one of the most widely used alveolar epithelial cell models, for our further experiments. To determine whether ZnONPs could induce cell death on respiratory epithelial cells, we treated A549 cells with 10, 30 and 100 *μ*g/ml ZnONPs. MTS results showed that treatment with ZnONPs induced significant cell death at doses of 30 and 100 *μ*g/ml (Figure 1a).

Dissolution of ZnONPs has been demonstrated as contributing to the cytotoxicity of ZnONPs. Hence, we examined the intracellular concentration of zinc ions using FluoZin-3, a specific indicator for zinc ions. Results of fluorescence-activated cell sorting (FACS) showed that ZnONPs induced marked elevations of zinc ions signal in A549 cells (Figure 1b). Meanwhile, the mRNA expression level of zinc transporter 1 (ZnT1) was also elevated (Supplementary Figure S2a). To determine whether the elevation of zinc ions concentration was correlated with cell death, zinc ions-chelating agent diethylenetriaminepentaacetic acid (DTPA) and *N*,*N*,*N*',*N*'-Tetrakis (2-pyridylmethyl) ethylenediamine (TPEN) were added 1 h before ZnONPs treatment. Results indicated that chelating of intracellular zinc ions alleviated A549 cell death induced by ZnONPs treatment at doses of 30 and 100 *μ*g/ml (Figure 1c). The discrepant rescue capacity between DTPA and TPEN might be due to the maximum-tolerated nontoxic dose (Supplementary Figures S2b and c). Moreover, DTPA could improve cell viability in a dose-dependent manner (Figure 1d), and might not via affecting the entry of ZnONPs (Supplementary Figure S2d). We also observed that DTPA treatment significantly reduced intracellular zinc ions concentration (Figure 1e). At the same time, we ruled out the possibility of apoptotic cell death after ZnONPs treatment; the evidence contributing to this conclusion included the following: (1) Annexin V-FITC/propidium iodide staining showed no significant apoptosis in ZnONPs-treated A549 cells (Supplementary Figure S3a); (2) ZnONPs treatment had minimal influence on the apoptosis marker including cleaved PARP and cleaved caspase-3 (Supplementary Figure S3b); and (3) pancaspase inhibitor Z-VAD-FMK could not rescue ZnONPs-induced cell death even at a high dose (Supplementary Figure S3c). Taken together, these data demonstrated that zinc ion is the key mediator in ZnONPs-induced non-apoptotic cell death.

### ZnONPs induce accumulation of autophagosomes and impairment of autophagic flux

**Figure 2:**
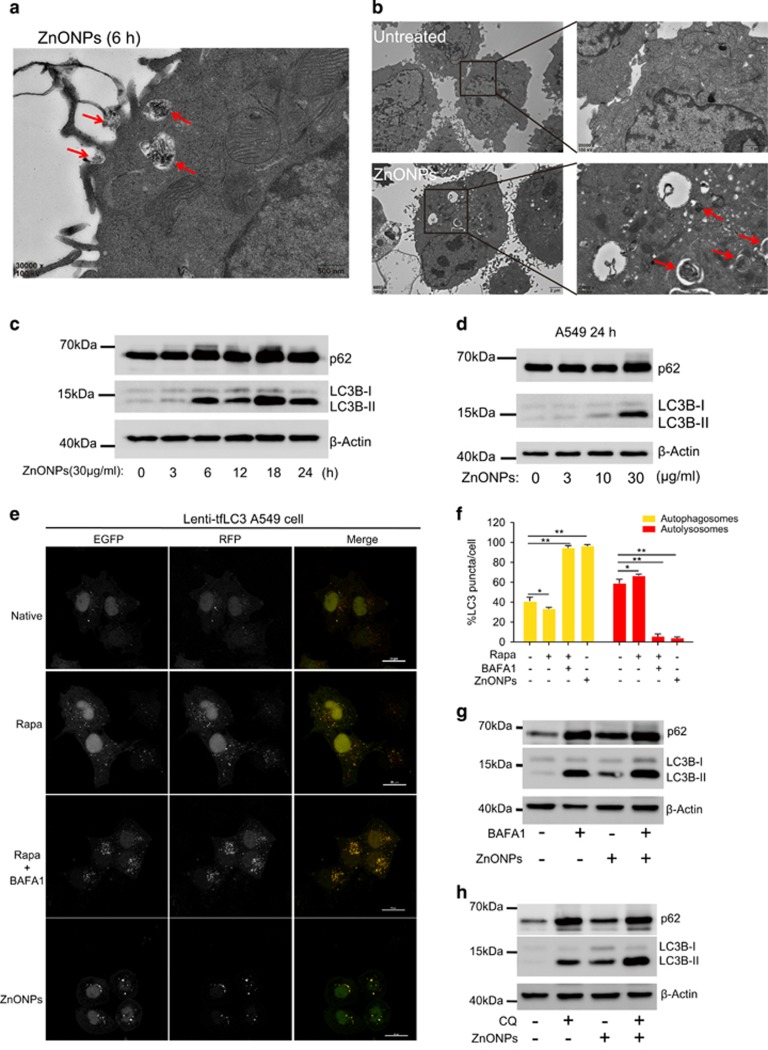
Autophagy is involved in ZnONPs-induced cytotoxicity. (**a**) TEM images of A549 cells treated with 30 *μ*g/ml ZnONPs at 6 h after treatment. Red arrows indicated that ZnONPs were wrapped into cells. Images are representative of three independent experiments. (**b**) TEM images of A549 cells at 0 or 24 h after treatment with 30 *μ*g/ml ZnONPs. Red arrows indicate autophagosomes induced by ZnONPs (scale bar, 1 *μ*m). Images are representative of three independent experiments. (**c**) Western blot analysis of LC3 and p62 expression level in A549 cells treated with 30 *μ*g/ml ZnONPs at an indicated time. Images are representative of three independent experiments. (**d**) Western blot analysis of LC3 and p62 expression levels in A549 cells treated with either 0, 3, 10 and 30 *μ*g/ml ZnONPs at 24 h after treatment. Images are representative of three independent experiments. (**e**) tfLC3 stably expressing A549 cells were treated either with Rapa (2 *μ*M), Rapa plus BAFA1 (100 nM) for 4 h or with 30 *μ*g/ml ZnONPs for 24 h. Confocal images were obtained after formalin fixation of cells (scale bar, 20 *μ*m). Images are representative of three independent experiments. (**f**) The number of yellow puncta (autophagosomes) and mRFP LC3-positive puncta (autolysosomes) in the merged images of (**e**) was counted and the total number of puncta per cell was calculated as the percentage. Data are representative of three independent experiments and values are expressed in mean±S.E.M., *P<0.05 and **P<0.01. (**g**) Western blot analysis of LC3 and p62 expression levels in A549 cells treated with either vehicle, BAFA1 (100 nM), ZnONPs (30 *μ*g/ml) or ZnONPs plus BAFA1 at 24 h after treatment. Images are representative of three independent experiments. (**h**) Western blot analysis of LC3 and p62 expression levels in A549 cells treated with vehicle, CQ (10 *μ*M), ZnONPs (30 *μ*g/ml) or ZnONPs plus CQ at 24 h after treatment. Images are representative of three independent experiments

Autophagy has been considered as an important event in nanoparticle-induced cytotoxicity. In particular, ZnONPs have been linked to autophagy. In line with pervious observations, our TEM results showed that ZnONPs were wrapped by cell plasma membrane and formed a phagophore-like structure at 6 h after treatment with ZnONPs (Figure 2a). Autophagosomes and autolysosomes in A549 cells increased markedly at 24 h after treatment with ZnONPs (Figure 2b). Meanwhile, western blot analysis results showed that the autophagy marker, LC3B-II/LC3B-I ratio, increased at 6 h and kept increasing during the treatment period with ZnONPs (Figure 2c) in a dose-dependent manner (Figure 2d). Interestingly, we also noted that p62 (SQSTM1/sequestome1), a specific substrate degraded by autophagy,^32^ increased in ZnONPs-treated cells and exhibited the same pattern as LC3B-II/LC3B-I ratio. These results indicated that ZnONPs might cause impairment of autophagic flux in A549 cells.

We confirmed this notion that the impairment of autophagic flux was induced by ZnONPs in transgenic cells stably expressing mRFP-GFP tandem fluorescent-tagged LC3 (tfLC3). Because GFP fluorescence was not stable in the acidic environment of lysosomes, tfLC3 showed a GFP and mRFP signal (merged as yellow puncta) before the fusion with lysosomes, and exhibited only the mRFP signal subsequently in autolysosomes.^33^ Therefore, autophagosomes and autolysosomes are labeled with yellow (i.e., mRFP and GFP) and red (i.e., mRFP only) signals, respectively.^34^ As shown in Figures 2e and f, autophagy inducer rapamycin (Rapa) induced more red puncta than untreated cells, suggesting an enhancement of autophagic flux. The specific inhibitor of vacuolar-type H^+^-ATPase bafilomycin A1 (BAFA1) could prevent the fusion of autophagosomes with lysosomes. By cotreating BAFA1 with Rapa, many yellow puncta were observed, suggesting that the autophagic flux was blocked. Consistent with the above results (Figures 2c and d), yellow puncta increased significantly in ZnONPs-treated A549 cells (Figures 2e and f), confirming that ZnONPs treatment indeed blocked autophagic flux. Autophagosome accumulation accompanied with the increased autophagy substrate could result from either autophagy induction or impairment of autophagic flux.^28^ To verify whether autophagy was induced upon ZnONPs treatment, A549 cells were pretreated with BAFA1 1 h before ZnONPs treatment. When lysosomal degradation is inhibited, the amount of LC3B-II is strictly dependent on LC3B-II production, and thus could reflect whether autophagy is induced.^35^ Western blot results showed that there were increases in LC3B-II and p62 levels in cells co-treated with ZnONPs and BAFA1, indicating that ZnONPs do indeed induce autophagy (Figure 2g). This phenomenon was further confirmed by treating cells with chloroquine diphosphate salt (CQ), which could elevate the lysosomal pH and impair the fusion of autophagosomes with lysosomes (Figure 2h). Collectively, our data demonstrate that ZnONPs not only induce autophagy but also impair the autophagic flux in alveolar epithelial cells.

### Inhibition of autophagy ameliorates cell death induced by ZnONPs

**Figure 3:**
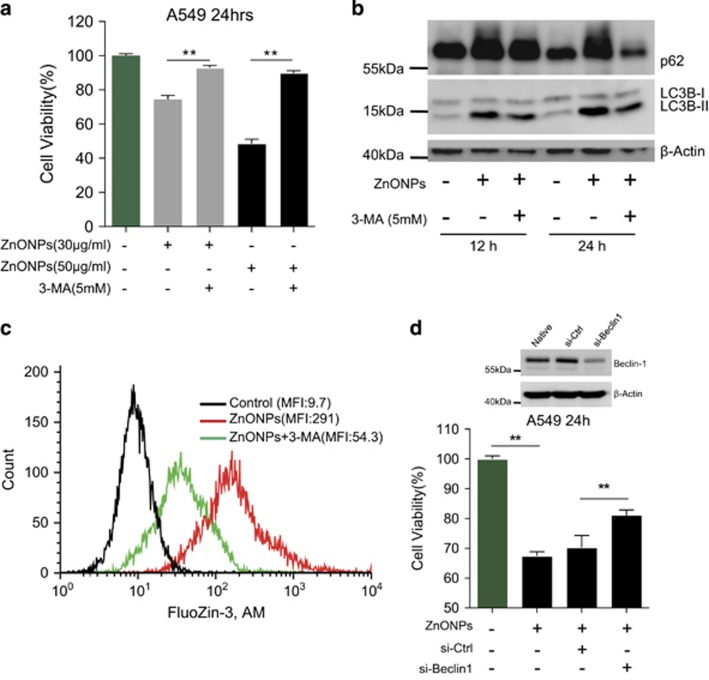
Inhibition of autophagy mitigates ZnONPs-induced cell death. (**a**) A549 cells were pretreated with 3-MA (5 mM), followed by treatment with 30 or 50 *μ*g/ml ZnONPs. MTS analysis of A549 cells was performed at 24 h after ZnONPs treatment. Data are representative of three independent experiments (*n*=6 for each group) and values are expressed as mean±S.E.M. **P<0.01. (**b**) Western blot analysis of LC3 and p62 expression levels in A549 cells treated with 3-MA (5 mM), followed by treatment with 30 *μ*g/ml ZnONPs at an indicated time. *β*-Actin served as a loading control. Images are representative of three independent experiments. (**c**) A549 cells were treated with 3-MA (5 mM), followed by treatment with 30 *μ*g/ml ZnONPs. FACS analysis of cells stained with FluoZin-3, AM was performed at 24 h after treatment. Images are representative of three independent experiments. (**d**) A549 cells were transfected with 75 nM control siRNA (si-Ctrl) or specific Beclin-1 siRNA (si-Beclin-1) for 48 h. The efficiency of knockdown was detected by western blot analysis. *β*-Actin served as a loading control. Images are representative of three independent experiments (top panel). MTS analysis was then performed to detect the cell viability of si-Ctrl- or si-Beclin-1-treated cells at 24 h after treatment with 30 *μ*g/ml ZnONPs. Data are representative of three independent experiments (*n*=6 for each group) and values are expressed as mean±S.E.M. **P<0.01 (bottom panel)

To clarify the relationship between autophagy induction and cytotoxicity mediated by ZnONPs, the PI3K inhibitors (3-methyladenine (3-MA) and wortmannin) were applied to inhibit autophagy.^36^ Cell viability increased significantly after pretreatment with 3-MA (Figure 3a) or wortmannin (Supplementary Figure S4a) in comparison with only ZnONPs treatment. Meanwhile, 3-MA inhibited ZnONPs-induced elevation of LC3B-II/LC3B-I ratio and p62 expression (Figure 3b), and reduced the intracellular concentration of zinc ions in ZnONPs-treated cells (Figure 3c). However, Rapa treatment seemed to have minimal influence on cell viability when A549 cells were treated with ZnONPs (Supplementary Figure S4b). Furthermore, siRNA-mediated knockdown of Beclin-1 (Atg6), which had a central role in autophagy,^37^ could rescue the cell viability in ZnONPs-treated cells (Figure 3d). In addition, DTPA treatment could significantly decrease the LC3B-II/LC3B-I ratio and p62 expression in ZnONPs-treated cells, which demonstrated that the release of zinc ions is certainly associated with autophagy (Supplementary Figure S4c). Collectively, our data demonstrate that autophagy induction is positively correlated with the elevation of zinc ions concentration and ZnONPs-induced cell death.

### AMPK pathway activation is involved in ZnONPs-induced A549 cell death

**Figure 4:**
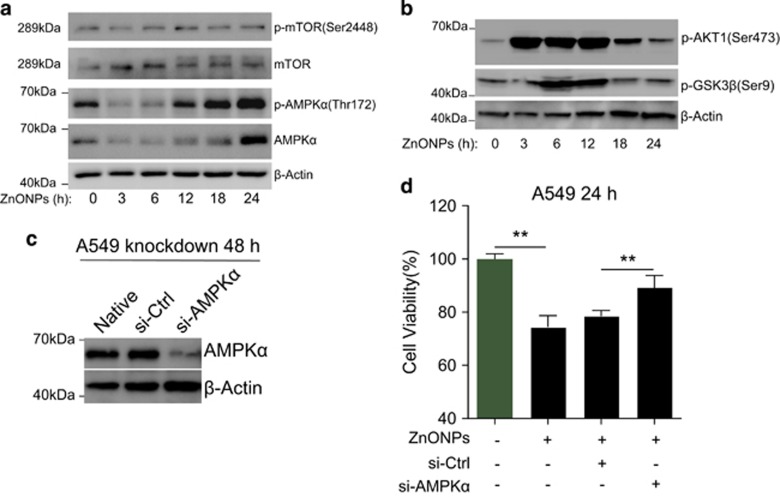
AMPK*α* contributes to ZnONPs-induced A549 cell death. (**a**) mTOR, p-mTOR, AMPK*α*, p-AMPK*α* and (**b**) p-AKT1, p-GSK3*β* expression levels in A549 cells treated with ZnONPs (30 *μ*g/ml) were determined by western blot analysis at an indicated time. *β*-Actin served as a loading control. Images are representative of three independent experiments. (**c**) A549 cells was transfected with 75 nM control siRNA (si-Ctrl) or AMPK*α* siRNA (si-AMPK*α*) for 48 h. The efficiency of knockdown was detected by western blot analysis. *β*-Actin served as a loading control. Images are representative of three independent experiments. (**d**) MTS analysis was then performed to detect the cell viability of si-Ctrl- or si-AMPK*α*-treated cells at 24 h after treatment with 30 *μ*g/ml ZnONPs. Data are representative of three independent experiments (*n*=6 for each group) and values are expressed as mean±S.E.M. **P<0.01

The inhibition of the target of Rapa (mTOR) and AMP-activated protein kinase (AMPK) activation are recognized as important regulatory mechanisms of autophagy.^38^ Intriguingly, we found that the p-mTOR level was not altered significantly upon ZnONPs treatment, whereas p-AMPK*α* expression level was markedly upregulated (Figure 4a). Meanwhile, we found that phosphorylation of protein kinase B/Akt, which was the upstream regulator of mTOR,^39^ significantly elevated after ZnONPs treatment (Figure 4b). p-AKT has been shown to rapidly phosphorylate glycogen synthase kinase 3 beta (GSK3*β*) at serine 9.^40^ We also detected that the p-GSK3*β*(Ser9) expression level was upregulated upon ZnONPs treatment (Figure 4b), further demonstrating that ZnONPs could induce the elevation of p-AKT. On the other hand, we performed siRNA-mediated knockdown of AMPK to investigate whether the correlation between AMPK*α* activation and ZnONP-induced cell death was an epiphenomenon, or whether they were causally linked. We observed that AMPK*α* knockdown (Figure 4c) caused slight elevation of the cell viability compared with control siRNA-treated cells upon ZnONP treatment (Figure 4d), suggesting that AMPK*α* activation might be involved in ZnONPs-induced cell death.

### Aberrant LAMP-2 expression contributes to impaired autophagic flux

Delivery of ZnONPs to lysosomes and dissolution of ZnONPs in lysosomes are supposed to be linked to lysosomal dysfunction,^13, 14^ which might contribute to the impairment of autophagic flux.

**Figure 5:**
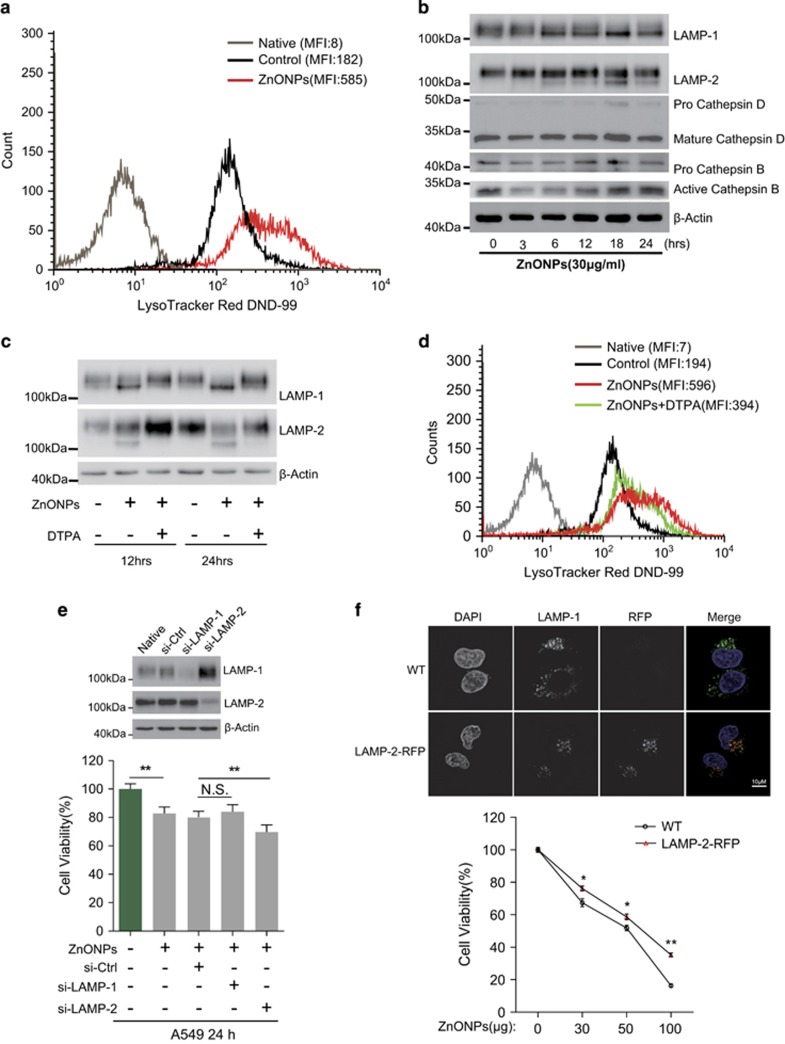
Dysfunction of lysosomes induced by ZnONPs. (**a**) FACS analysis of cells stained with LysoTracker Red DND-99. The effect of vehicle and ZnONPs (30 *μ*g/ml) treatment on lysosomes amount was analyzed 24 h after treatment. Images are representative of three independent experiments. (**b**) Western blot analysis of LAMP-1, LAMP-2, cathepsin B and cathepsin D expression levels in A549 cells treated with ZnONPs (30 *μ*g/ml) at an indicated time. *β*-Actin served as a loading control. Images are representative of three independent experiments. (**c**) A549 cells were pretreated with DTPA (1 mM), followed by treatment with ZnONPs (30 *μ*g/ml). Western blot analysis of LAMP-1 and LAMP-2 expression levels was performed at 12 or 24 h after treatment. *β*-Actin served as a loading control. Images are representative of three independent experiments. (**d**) A549 cells were pretreated with DTPA (1 mM), followed by treatment with ZnONPs (30 *μ*g/ml). FACS analysis of cells stained with LysoTracker Red DND-99 was performed to detect the amount of lysosomes at 24 h after treatment. Images are representative of three independent experiments. (**e**) A549 cells were transfected with 75 nM siRNA against LAMP-1, LAMP-2 or control siRNA for 48 h. Western blot analysis was performed to verify the knockdown efficiency. Images are representative of three independent experiments (top panel). Then, cells were treated with 30 *μ*g/ml ZnONPs, and MTS analysis was performed to detect the cell viability at 24 h after treatment with 30 *μ*g/ml ZnONPs. Data are representative of three independent experiments (*n*=6 for each group) and values are expressed in mean±S.E.M. **P<0.01 (bottom panel). (**f**) The wild-type (WT) A549 cell line and the A549 cell line stably expressing LAMP-2-RFP were determined by immunofluorescence. Note that only LAMP-2-RFP cells represented red signal and had colocalization with LAMP-1, indicating correct expression of LAMP-2-RFP. Images are representative of three independent experiments. Scale bar=10μm (top panel). WT and LAMP-2-RFP cells were treated with ZnONPs at 30, 50 or 100 *μ*g/ml. MTS analysis was performed 24 h after ZnONPs treatment. Data are representative of three independent experiments (*n*=6 for each group) and values are expressed as mean±S.E.M. *P<0.05 and **P<0.01 (bottom panel)

To determine how the blockade of autophagic flux was induced in ZnONPs-treated cells, we labeled the cells with LysoTracker Red DND-99, a probe accumulated within the acidic lysosomes/late endosomes. FACS results show that treatment with ZnONPs increased the fluorescence signal of LysoTracker Red DND-99, suggesting that the amount of acidic lysosomes increased (Figure 5a). Immunofluorescence results showed that the signal intensity of LAMP-1/2 was slightly diffused in ZnONPs-treated cells (Supplementary Figure S5a). Interestingly, western blot assay data showed that cathepsin B/D had only a slight change, whereas aberrant LAMP-1/2 expression occurred upon ZnONPs treatment (Figure 5b). Furthermore, we observed that DTPA could ameliorate aberrant LAMP-1/2 expression (Figure 5c) and decrease the amount of lysosomes in cells treated with ZnONPs (Figure 5d), suggesting that zinc ions induced the activation of lysosomes and the aberrant LAMP-1/2 expression. To determine whether LAMPs were involved in ZnONPs-induced cell death, knockdown of LAMP-1/2 was performed before treatment with ZnONPs. Western blot analysis results showed a decrease in LAMP-1 and LAMP-2 expression (Figure 5e, top panel). Further, LAMP-1 or LAMP-2 knockdown had no significant influence on naïve autophagy level (Supplementary Figure S5b) and A549 cell viability (Supplementary Figure S5c). Interestingly, LAMP-2 knockdown, but not LAMP-1 knockdown, increased the ZnONPs-induced cell death (Figure 5e, bottom). More importantly, we observed that the A549 cell line stably expressing LAMP-2-RFP (Figure 5f, top panel) was more resistant than the wild-type A549 cell line with ZnONPs treatment (Figure 5f, bottom panel). We also observed that LAMP-2 expression level was elevated in the Rapa-induced autophagy model (Supplementary Figures S5d and e).

We also investigated additional mechanisms that could be responsible for ZnONPs-induced impairment of autophagic flux. Lysosomal membrane permeabilization, a process induced by the damage of lysosomal membrane integrity, could lead to the leakage of hydrolytic enzyme proteases, such as cathepsin B, D and L, from lysosomal lumen to cytosol.^41, 42^ However, our immunofluorescence data showed that cathepsin D still colocalized with LAMP-1 after ZnONPs treatment (Supplementary Figure S6a) rather than diffused through the entire cell, which was a typical feature during lysosomal membrane permeabilization.^43^ siRNA-mediated knockdown of cathepsin B, one of the most important enzymes in lysosomal membrane permeabilization,^44^ did not ameliorate ZnONPs-induced cell death (Supplementary Figure S6b). Treatment with Z-VAD-FMK, which was reported to block the activity of lysosomal proteases, did not alleviate ZnONPs-induced cell death (Supplementary Figure S3c). These data indicated that the impairment of autophagic flux might not result from ZnONPs-induced lysosomal membrane permeabilization. In addition, lysosomal alkalinization is regarded as the major reason for the blockade of autophagic flux in AuNP-treated NRK cells.^35^ We stained A549 cells with LysoSensor Green DND-189, an acidotropic dye, which could be used to measure the pH of acidic lysosomes. The FACS data showed that ZnONPs treatment could not affect lysosomal acidic environment in A549 cells (Supplementary Figure S6c).

### Impaired autophagic flux results in the accumulation of damaged mitochondria and excessive ROS production

ROS, generated mainly as by-products of mitochondrial respiration,^45^ are increasingly identified as a major contributor to the cytotoxicity induced by nanoparticles, including ZnONPs.^8^ Mitophagy is triggered for effective removal of damaged mitochondria and is implicated in diminishing excessive ROS.^28^ On the other hand, previous studies have reported that LAMP-2 is involved in the fusion of phagosomes with lysosomes,^46, 47^ and thus it could contribute to the mitophagy regulation.^48^ On the basis of these studies and our experimental data, we presume that damaged mitochondria fail to be eliminated as ZnONPs treatment causes aberrant LAMP-2 expression and sequential mitophagy dysfunction.

**Figure 6:**
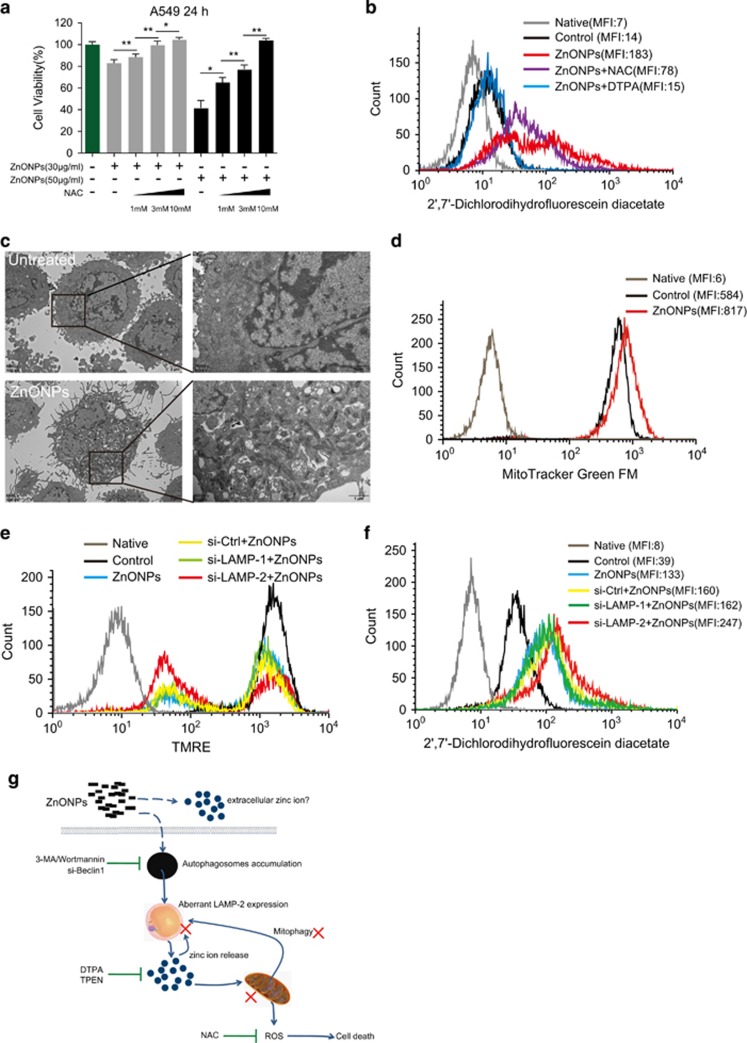
ZnONPs induce excessive ROS production resulting from the accumulation of damaged mitochondria. (**a**) A549 cells were pretreated with 1, 3 or 10 mM NAC, followed by 30 or 50 *μ*g/ml ZnONPs treatment. MTS analysis was performed to detect the cell viability at 24 h after treatment. Data are representative of three independent experiments (*n*=6 for each group) and values are expressed in mean±S.E.M. *P<0.05, **P<0.01. (**b**) A549 cells were pretreated with NAC (10 mM) or DTPA (1 mM), followed by treatment with 30 *μ*g/ml ZnONPs. FACS analysis of cells stained with 2'7'-dichlorofluorescin diacetate was performed at 24 h after treatment. Images are representative of three independent experiments. (**c**) TEM images of untreated or 30 *μ*g/ml ZnONPs-treated A549 cells. Note that aberrant mitochondria accumulated in ZnONPs-treated cells. Images are representative of three independent experiments. (**d**) FACS analysis of cells stained with MitoTracker-Green FM. The effects of vehicle or ZnONPs (30 *μ*g/ml) on the mass of mitochondria were analyzed 24 h after treatment. Images are representative of three independent experiments. (**e**) A549 cells were transfected with 75 nM siRNA targeted to LAMP-1, LAMP-2 or control siRNA for 48 h. Thereafter, cells were treated with 30 *μ*g/ml ZnONPs, and FACS analysis of cells stained with TMRE was performed to detect mitochondrial membrane potential at 24 h after treatment. Images are representative of three independent experiments. **P<0.01, N.S., not significant. (**f**) A549 cells were transfected with 75 nM siRNA targeted to LAMP-1, LAMP-2 or control siRNA for 48 h. Then, cells were treated with 30 *μ*g/ml ZnONPs and FACS analysis of cells stained with 2'7'-dichlorofluorescin diacetate was performed to detect intracellular ROS at 24 h after treatment. Images are representative of three independent experiments. (**g**) Schematic of the mechanism of ZnONPs-induced cytotoxicity. ZnONPs are delivered into lysosomes via autophagy, and subsequently dissolved in lysosomes to release zinc ions, which is the crucial factor triggering ZnONPs cytotoxicity. Furthermore, zinc ions (intracellular or extracellular) can damage mitochondria and lysosomes; thereafter, they further disrupt the negative feedback mechanisms of ROS and mitophagy, leading to the accumulation of damaged mitochondria, excessive ROS and finally cell death. Aberrant LAMP-2 expression is probably involved in this process. Inhibition of autophagy, chelating of zinc ions or treatment with antioxidant NAC effectively mitigates ZnONPs-induced cytotoxicity

To verify our hypothesis, we labeled ZnONPs-treated cells with 2'7'-dichlorofluorescin diacetate to detect the intracellular ROS level. The FACS data showed that ZnONPs caused significant elevation in the ROS level (Supplementary Figure S7a). Furthermore, pretreatment with *N*-acetyl-l-cysteine (NAC), a well-known antioxidant and free radical scavenger, ameliorated ZnONPs-induced cell death in a dose-dependent manner (Figure 6a). The pretreatment with NAC or DTPA cleared out excessive ROS after ZnONPs treatment (Figure 6b). Furthermore, NAC could decrease the LC3B-II/I ratio, p62 and aberrant LAMP-1/2 expression with ZnONPs treatment (Supplementary Figure S7b), suggesting the interplay between ROS and autophagy. These data collectively demonstrate that ZnONPs treatment could induce ROS production and trigger cell death in a ROS-dependent manner.

To determine whether ZnONPs treatment induced mitochondrial damage, we stained A549 cells with tetramethylrhodamine ethyl ester perchlorate (TMRE), a potential-sensitive probe for measuring membrane potential changes in the mitochondria. FACS results showed that ZnONPs treatment decreases the fluorescence signal of TMRE (Supplementary Figure S7c), indicating that the mitochondria were damaged. On the other hand, TEM images showed the accumulation of aberrant mitochondria in ZnONPs-treated cells compared with untreated cells (Figure 6c). Moreover, we labeled cells with MitoTracker-Green FM, which stains mitochondria regardless of the mitochondrial membrane potential and only reflects the number of mitochondria. FACS results showed an increased fluorescence signal in ZnONPs-treated cells, indicating that ZnONPs treatment caused mitochondrial accumulation in A549 cells (Figure 6d). To investigate whether LAMPs were involved in the regulation of mitophagy and ROS in ZnONPs-treated cells, we performed siRNA-mediated knockdown of LAMP experiments. FACS data showed that LAMP-2 knockdown significantly increased the ratio of cells with low signal of TMRE (Figure 6e) and evoked excessive ROS generation (Figure 6f). However, LAMP-1 knockdown seemed not to be responsible for the removal of damaged mitochondrial accumulation and excessive ROS generation.

In conclusion, we demonstrated that ZnONPs-induced cell death resulted from the cascade of autophagic flux impairment following damaged mitochondrial accumulation and excessive ROS production. This process might attribute to aberrant LAMP-2 expression induced by zinc ions released from ZnONPs in lysosomes. AMPK*α* activation, but not inhibition of mTOR, was involved in ZnONPs-induced cell death. The inhibition of autophagy, chelating of intracellular zinc ions using DTAP/TPEN or antioxidant NAC treatment can effectively mitigate ZnONPs-induced cytotoxicity (Figure 6g).

## Discussion

Previous studies have extensively investigated the toxicity effect of ZnONPs and have proposed potential regulatory mechanisms including ROS production and autophagy induction. However, the detailed regulatory mechanism of autophagy and, more importantly, the interplay between autophagy and ROS in ZnONPs-treated cells are still largely obscure. Our results indicated that ZnONPs subtly harness the two important functions of autophagy in lung epithelial cells: foreign material engulfment and damaged organelles elimination.

First, we demonstrated that ZnONPs induced autophagy, which facilitated ZnONPs to be delivered into lysosomes. The acidic environment of lysosomes would enhance ZnONPs dissolution and the sequential release of zinc ions.^13^ The high concentration of zinc ions directly induced cell death, which could be ameliorated by zinc-specific chelating agents such as DTPA or TPEN. Although TPEN was toxic at a concentration higher than 1 *μ*M alone (Supplementary Figure S2c), its synergistic treatment with ZnONP might be quenched, and thus TPEN could be applied at a concentration higher than 1 *μ*M. Our results showed that DTPA inhibited ZnONPs-mediated cell death and abrogated intracellular zinc ions; however, this did not rule out chelation of free zinc ions from ZnONPs upon addition to the culture media. Furthermore, we demonstrated that inhibition of autophagy could reduce the release of zinc ions and ameliorate ZnONPs-induced cell death. These results manifested that ZnONPs could use autophagy to release zinc ions and consequently induce non-apoptotic cell death.

More interestingly, we demonstrated that ZnONPs treatment could induce impaired autophagic flux. We observed that the p62 expression level was upregulated during the process of ZnONPs treatment, suggesting the impaired autophagic flux caused by ZnONPs treatment. This notion was further confirmed in the mRFP-GFP-LC3 stably expressing cell line. We found that a significant decrease of red-only puncta and a significant increase of yellow puncta occurred upon ZnONPs treatment, which is the sign of impaired lysosome degradation. Lin *et al.*^49^ showed that ZnONPs could upregulate the expression level of both LC3II and p62 in the human embryonic kidney cell line HEK-293, but they did not further describe the consequent biological effect induced by impaired autophagic flux. Several studies have discussed the relationship between ZnONPs and autophagy in T cells (SupT1 and Jurkat cell lines, primary human T cells),^27^ mouse macrophages,^16^ and JB6 Cl 41-5a mouse skin epidermal normal cells.^26^ However, they did not mention the phenomenon that caused impairment of autophagic flux induced by ZnONPs treatment. To our knowledge, the current study is the first to describe the impaired autophagic flux induced by ZnONPs in lung epithelial cells.

We further explored the possible mechanisms behind the impaired autophagic flux caused by ZnONPs. Unexpectedly, we found that the inhibition of mTOR, a negative regulator of autophagy, was probably not involved in ZnONPs-induced lung epithelial cell death. In contrast, Roy *et al.*^16^ reported that ZnONPs could induce apoptosis by enhancement of autophagy via PI3K/AKT/mTOR inhibition in mouse macrophages.^16^ Their results showed that ZnONPs reduced the phosphorylation levels of PI3K, AKT and mTOR in a time-dependent manner.^16^ However, numerous lines of evidence have shown that zinc ions could rapidly increase the phosphorylation levels of AKT.^50, 51, 52, 53^ Our results would support the later opinion as we observed that ZnONPs induced rapid increase of p-AKT (Ser473) and its substrate p-GSK*β* (Ser9) (Figures 4c and d). We suspect that the rapid and strong increase of p-AKT could preclude the inhibition of mTOR from induction of autophagy in ZnONPs-treated lung epithelial cells.

In addition to the inhibition of mTOR, AMPK activation is considered as another crucial event in autophagy regulation.^54, 55, 56^ We observed a strong time-dependent increase in p-AMPK*α* with ZnONPs exposure. AMPK*α* knockdown experiments further demonstrated the association between AMPK activation and cell death upon ZnONP treatment. AMPK activity can be regulated by AMP/ATP ratios, liver kinase B1, Ca^2+^/calmodulin-dependent protein kinase or TGF-*β*-activated kinase 1.^57, 58^ More relevantly, Yu *et al.*^26^ showed that ZnONPs induced an increase in total AMPK and a decrease in the relative ATP amount in normal mouse skin epidermal cells.^26^ Although evidence of AMPK*α* activation could not be accounted for, we showed that AMPK*α* activation partially contributed to ZnONPs-induced cell death.

In addition, we found that ZnONPs caused aberrant LAMP-1/2 expression. LAMP-2 knockdown or overexpression experiments demonstrated that LAMP-2 was linked to ZnONPs-induced ROS generation and cell death. Our previous study demonstrated that extensive LAMP-1/2 deglycosylation could induce lysosomal membrane permeabilization and cell death upon influenza virus infection.^59^ Moreover, recent studies suggested that sugar modification could contribute to the regulation of autophagosomes–lysosomes fusion.^60, 61^ Therefore, the existence of LAMP-2 deglycosylation and its potential roles in ZnONPs-induced impaired autophagic flux need be further explored.

In summary, we demonstrated that ZnONPs could induce autophagy to facilitate their dissolution in lysosomes, while inhibition of autophagy could decrease intracellular zinc ions concentration and ameliorate cell death. AMPK*α* activation was involved in ZnONPs-induced cell death. We also showed that ZnONPs could cause impaired autophagic flux, which could lead to ROS production. LAMP-2 appeared to contribute to impaired autophagic flux and to accumulation of damaged mitochondria and cell death in ZnONPs-treated lung epithelial cells. Our study provides a novel insight into the regulation mechanisms of autophagy–lysosomes–mitochondria–ROS axis, which would contribute to a better understanding of the toxicity of nanomaterials.

## Materials and methods

### Nanoparticles

Zinc oxide nanopowder was obtained from Sigma-Aldrich (St. Louis, MO, USA) (677450). The particle size of ZnONPs was ⩽50 nm (BET data provided by Sigma-Aldrich), which was confirmed by the supplier using X-ray diffraction. We also detected these ZnONPs using TEM, and the results are provided in Supplementary Figure S1a. ZnONPs were suspended in culture medium at a concentration of 3 mg/ml and then sonicated in a sonicator bath for 30 min. The solution was then diluted with a medium to a indicated concentration. The dilutions of ZnONPs were vigorously vortexed for 30 s before cell exposure to avoid nanoparticle agglomeration.

### Reagents

DTPA (D6518), TPEN (P4413), 3-MA (M9281), CQ (C6628) and NAC (A9165) were obtained from Sigma-Aldrich. BAFA1 (sc-201550) was purchased from Santa Cruz (Santa Cruz, CA, USA). Wortmannin (S2758), Rapa (S1039) and Z-VAD-FMK (S7023) were purchased from Selleck (Houston, TX, USA). The human lung adenocarcinoma A549 cell line was purchased from the American Type Culture Collection (ATCC, Rockville, MA, USA) and was cultured in RPMI-1640 medium (Gibco) supplemented with 10% fetal bovine serum (Gibco), 100 U of penicillin/ml and 100 U of streptomycin/ml at 37 °C with 5% CO_2_.

### Cloning and generation of stable cell lines

ptf-LC3 was a gift from Tamotsu Yoshimori (Addgene, Cambridge, MA, USA; plasmid no. 21074).^33^ LAMP-2A (NM_002294.2) was amplified from a human cDNA library and fused with RFP tag. The mRFP-EGFP-LC3 fragment in ptf-LC3 and the LAMP-2A-RFP fragment were subcloned into the lentiviral expression plasmid pCDH-CMV-MCS-EF1-Puro from System Biosciences (Palo Alto, CA, USA), respectively.

Lentiviruses were packaged in HEK293T cells. To generate stably transfected cell lines, A549 cells were infected with virus for 48 h and then selected using puromycin (Solarbio, Beijing, China).

### Transmission electron microscopy

The morphology of A549 cells was observed by TEM as described previously.^62^ Briefly, cells were centrifuged at 1000 × *g* for 5 min after trypsinization, fixed with 4% glutaraldehyde in 0.1M PBS (pH 7.4) for 2 h at 4 °C, washed three times with PBS (pH 7.4) and post-fixed with 1% osmium tetroxide in 0.1 M PBS for 1 h at 4 °C, dehydrated in a graded series of alcohol and acetone, and then embedded in Epon 816. The ultrathin sections were obtained using a Leica ultramicrotome (Leica Microsystems, Buffalo Grove, IL, USA). The ultrathin sections stained with uranyl acetate and lead citrate were examined with a Hitatch-7500 transmission electron microscope (Hitachi, Tokyo, Japan). For ZnONPs TEM detection, ZnONPs were suspended in deionized water at a concentration of 3 mg/ml and then sonicated in a sonicator bath for 60 min. The solution was then diluted with deionized water to a concentration of 30 *μ*g/ml. The dilutions of ZnONPs were vigorously vortexed for 5 min before TEM detection to avoid nanoparticle agglomeration.

### Cell viability assays

Cell viability was determined by the MTS assay (Promega, Madison, WI, USA) as described previously.^63^ In the rescue experiments, DTPA, TPEN, 3-MA, Rapa, wortmannin and NAC were added 1 h before, and for the duration of, the stimulation, or at the indicated time points shown in the figure legend. In the siRNA knockdown experiment, A549 cells were transfected with siRNA as described previously, and the MTS assay was performed at 24 h after treatment with nanomaterials.

### FACS analysis

For zinc concentration detection, A549 cells were seeded into 6-well plates. After 24 h of ZnONPs treatment, zinc-specific indicator FluoZin-3, AM (Molecular Probe, Waltham, MA, USA) was added to the cell at a final concentration of 500 nM, followed by 30 min incubation at room temperature in the dark. Thereafter, cells were washed three times using the complete medium, followed by an additional incubation for 30 min at room temperature in the dark to allow complete de-esterification of intracellular AM esters.

For ROS detection, 2',7'-dichlorofluorescin diacetate (Sigma-Aldrich) was diluted in FBS-free medium to 20 *μ*M and then added to the cells, followed by 30 min incubation at 37 °C.

For lysosome detection, LysoTracker DND-99 (Molecular Probe) and LysoSensor Green DND-199 (Molecular Probe) were diluted in prewarmed complete medium to 50 nM and 1 *μ*M separately, and then were added to cells, followed by 30 min incubation at 37 °C.

For measuring membrane potential changes in the mitochondria, TMRE (Sigma-Aldrich) was diluted in prewarmed FBS-free medium to a final concentration of 10 nM, and then was added to cells, followed by 20 min incubation at 37 °C. For measuring the mitochondrial mass, MitoTracker-Green FM (Molecular Probe) was diluted in prewarmed FBS-free medium to a final concentration of 100 nM, and then was added to cells, followed by 20 min incubation at 37 °C.

For apoptosis detection, the Annexin V-FITC/Propidium Iodide Apoptosis Detection Kit (Beyotime Biotechnology, Jiangsu, China) was used according to the manufacturer's instructions.

All FACS experiments were performed on BD Influx Cell Sorter (BD Biosciences, San Jose, CA, USA) and results were analyzed using the BD FACS Software (San Jose, CA, USA).

### Western blotting

For western blotting analysis, A549 cells were collected at an indicated time after treatment. Cells were washed two times with precold PBS and then treated with ice-cold RIPA lysis buffer (Abcam, Cambridge, MA, USA) containing PMSF (Sigma-Aldrich) and protease inhibitors (Thermo Fisher, Waltham, MA, USA). The protein concentrations of cell lysates were determined by the BCA Assay Kit (Thermo Fisher). Proteins were separated by SDS-PAGE, and then transferred on the PVDF membrane (EMD Millipore, Hayward, CA, USA). After blocking with 5% nonfat milk in PBS-Tween 20, the PVDF membrane was incubated with specific primary antibodies at 4 °C overnight, respectively. The primary antibodies used were as follows: LAMP-1 (D2D11), Beclin-1 (D40C5), AMPK*α* (D63G4), p-AMPK*α* (Thr172) (40H9) were purchased from Cell Signaling Technology (Danvers, MA, USA), LAPM-2 (H4B4) was purchased from Santa Cruz, p62/SQSTM1 (EPR4844), LC3B (EPR18709), cathepsin D (EPR3057Y), PARP (EPR18461), caspase-3 (EPR18297), mTOR (EPR390(N)), p-mTOR (EPR426(2)), p-AKT (EP2109Y) and p-GSK3*β* (EPR2286Y) were purchased from Abcam, cathepsin B(IM27L) was purchased from EMD Millipore and *β*-actin (BA3R) was purchased from Thermo Fisher. Finally, PVDF membranes were incubated with horseradish peroxidase-conjugated secondary antibodies (MultiSciences, Zhejiang, China) at room temperature for 1 h. Chemiluminescence immunodetection was performed with Immobilon Western Chemiluminescent HRP Substrate (EMD Millipore).

### siRNA transfection

All siRNAs used in this study were synthesized by GenePharma (Shanghai, China). Before siRNA transfection, A549 cells were seeded in 12-well plates. After 24 h, cells were transfected with siRNA duplexes (75 nM) with Lipofectamine RNAiMAX reagent (Invitrogen, Waltham, MA, USA) diluted in Opti-MEM (Invitrogen) according to the manufacturer’s instructions. Next, at 48 h after siRNA transfection, efficiency of knockdown was determined by western blotting.

The siRNA sequences were listed as follows:

si-Beclin-1: 5′-GCUGCCGUUAUACUGUUCUtt-3′^64^

si-LAMP-1: 5′-CAAUGCGAGCUCCAAAGAAtt-3′^59^

si-LAMP-2: 5′-GCGGUCUUAUGCAUUGGAAtt-3′^59^

si-Cathepsin B: 5′-GAGUUAUGUUUACCGAGGAtt-3′^65^

si-AMPK*α*: 5′-AAAUUCACCAUCUGACAUCAU-3′^66^

negative control siRNA: 5′-UUCUCCGAACGUGUCACGUtt-3′.

### Real-time quantitative PCR analysis

Total RNA was extracted from A549 cells using Eastep Super Total RNA Extraction Kit (Promega), cDNA was synthesized from 1.5 *μ*g of total RNA with GoScript Reverse Transcription Kit (Promega) according to the standard protocol provided by the manufacturer. PCR amplification assays were performed with GoTaq Real-time Quantitative PCR Master Mix (Promega) on CFX96 Touch Real-Time PCR Detection System (Bio-Rad, Hercules, CA, USA).The relative gene expression levels were calculated using the CT value and normalized to the expression of the TATA-binding protein (TBP) reference gene. The specific primers were synthesized by Sangon (Shanghai, China) and the sequences were as follows:

ZnT1 forward, 5′-TGTTGAAGGAGTTGAGGAA-3′

ZnT1 reverse, 5′-TGAATGGTAGTAGCGTGAA-3′

TBP forward, 5′-ATCAGTGCCGTGGTTCGT-3′

TBP reverse, 5′-TTCGGAGAGTTCTGGGATTG-3′.

### Confocal microscopy

Native A549 cells or A549 cells stably expressing mRFP-GFP tfLC3 were grown on coverslips in 24-well plates. At the indicated time points after NP treatment or stimulation, the coverslips were washed two times with PBS and fixed with 4% paraformaldehyde for 15 min at room temperature. After washing two times with PBS, the cells were permeabilized and blocked with 3% BSA and 0.1% Triton X-100 in PBS for 40 min at room temperature. The coverslips were then incubated with the indicated primary antibody at 4 °C overnight and then incubated with Alexa Fluor 488- or Alexa Fluor 568-labeled secondary antibody (Molecular Probes) together with DAPI (Molecular Probes) at room temperature for 1 h. The subcellular localization of each target protein was observed using confocal laser scanning microscopy (Nikon, Tokyo, Japan), and the images were analyzed using NIS-Elements Viewer 4.20 (Nikon, Tokyo, Japan). The primary antibodies used were as follows: LAMP-1 (ab24170; Abcam), LAMP-2 (H4B4; Santa Cruz), cathepsin D (E-7; Santa Cruz).

### Statistics analysis

All data are shown as means±S.E.M. Measurements at single time points were analyzed by analysis of variance, and in case of significance they were further analyzed by a two-tailed *t*-test. All statistical tests were conducted using Prism 5.0 (GraphPad Software, San Diego, CA, USA). *P<0.05 indicates statistical significance, **P <0.01 indicates high significance, and NS indicates no significance.
