# Supplementary material for: Zinc oxide nanoparticles harness autophagy to induce cell death in lung epithelial cells
Source: Cell Death Dis. 2017 Jul 27;8(7):e2954–. doi: 10.1038/cddis.2017.337 (PMC5550878; doi:10.1038/cddis.2017.337)
Supplement: Supplementary Figures Legends [file cddis2017337x1.docx]

**Supplementary Figure Legends**

**Figure S1 Characterization of ZnONPs**

ZnONPs was characterized by TEM scan. The particle size of ZnONPs was about or smaller than 50nm, and ZnONPs exhibited roughly sphericalmorphology.

**Figure S2 ZnONPs induce cell death via release of zinc ions**

**(a)** QPCR analysis was performed to detect ZnT1 mRNA relative expression level after treatment with vehicle orZnONPs(30μg/ml) at 24h after treatment in A549 cells. TBP served as reference gene. Data are representative of three independent experiments (n=3 for each group)and values are expressed in mean ± SEM.^**^*p*< 0.01. **(b)**MTS analysis was performed to detect A549 cells viability after treatment with DTPA at dose of 0, 0.1, 0.3, 1 and 3mM for 24h. Data are representative of three independent experiments (n=6 for each group)and values are expressed in mean ± SEM.^**^*p*< 0.01. **(c)**MTS analysis was performed to detect A549 cells viability after treatment with TPEN at dose of 0, 0.1, 0.3, 1 and 3μM for 24h. Data are representative of three independent experiments (n=6 for each group)and values are expressed in mean ± SEM.^**^*p*< 0.01. **(d)** MTS analysis was performed to detect cells viability of A549 cells treated with DTPA (1mM) 1h before, or treated 1 and 3hafter treatment withb ZnONPs (30μg/ml) for 24h. Data are representative of three independent experiments (n=6 for each group)and values are expressed in mean ± SEM.^**^*p*< 0.01.

**Figure S3 ZnONPs induce non-apoptotic cell death**

**(a)**FACS analysis of cells stained with Annexin V and PI. The effect of vehicle or ZnONPs(30μg/ml)treatment on apoptosis was analyzed 24h after treatment. Note Q2 and Q3 indicated apoptotic cells. Images are representative of three independent experiments. **(b)**Western-blot analysis of PARP, cleaved PARP, Caspase 3 and cleaved Caspase 3 expression levels in A549 cells treated with 30μg/ml ZnONPs at 24h after treatment. β-Actin served as loading control. Images are representative of three independent experiments. **(c)**A549 cells were pretreated with Z-VAD-FMK at dose of 0, 50 and 200μM then followed by ZnONPs (30μg/ml) treatment. MTS analysis was performed to detect cell viability at 24 hafter treatment. Data are representative of three independent experiments (n=6 for each group)and values are expressed in mean ± SEM.^**^*p*< 0.01. N.S.=Not significant.

**Figure S4 Autophagy is activated upon ZnONPs treatment**

**(a)** A549 cells were pretreated with wortmannin(2.5μM) followed by treatment with ZnONPs(30μg/ml). MTS analysis was performed to detect cell viability at 24 h after treatment. Data are representative of three independent experiments (n=6 for each group)and values are expressed in mean ± SEM.^**^*p*< 0.01. **(b)** A549 cells were pretreated with DMSO or Rapamycin(2μM) then followed by treatment with ZnONPs(30μg/ml). MTS analysis was performed to detect cell viability at 24 h after treatment. Data are representative of three independent experiments (n=6 for each group)and values are expressed in mean ± SEM. N.S.=Not significant. **(c)** A549 cells were pretreated with DTPA(1mM) followed by treatment with ZnONPs(30μg/ml).Western-blot analysis of p62 and LC3B expression levels were performed at 12 h and at 24h after treatment. β-Actin served as loading control. Images are representative of three independent experiments.

**Figure S5 LAMP-2 is linked to ZnONPs-induced toxicity**

**(a)** The effect of ZnONPs(30μg/ml for 24h) on LAMP-1 and LMAP-2 was detected by confocal microscope. Nuclei were counterstained with DAPI. Images are representative of three independent experiments. **(b)** A549 cells were transfected with 75nM siRNA targetedto LAMP1, LAMP2 or control siRNA for 48h. Then cells were collected for western-blot analysis to detect p62 and LC3B expression levels. β-Actin served as loading control. Images are representative of three independent experiments. **(c)** A549 cells were transfected with 75nM siRNA targeting LAMP1, LAMP2 or control siRNA for 48h then followed by MTS analysis. Data are representative of three independent experiments (n=6 for each group) and values are expressed in mean ± SEM. N.S.=Not significant. **(d)**A549 cell lysates were collected at 0, 2 and 4 h after treatment with Rapamycin (2μM). Western-blot analysis was performed to detect the expression level of p-mTOR, p62 and LAMP-2.β-Actin served as loading control.Note that Rapamycin induced dramatic downregulation of p-mTOR and p62. Images are representative of three independent experiments. **(e)**Statistical analysis of the relative band density ratio of LAMP-2 to β-Actin was performed usinga t-test. Error bars represent the SD of three independent experiments. ^**^*p*< 0.01.

**Figure S6 Lysosomal membrane permeabilization might not be involved in ZnONPs-induced cell death**

**(a)** The effects of ZnONPs(30μg/ml for 24h) on colocalization of LAMP1 and Cathepsin D were detected by confocal microscope. Nuclei were counterstained with DAPI. Images are representative of three independent experiments. **(b)**A549 cells were transfected with 75nM siRNA against Cathepsin B or control siRNA for 48h. Western-blot analysis was performed to verify the knockdown efficiency, images are representative of three independent experiments(Top panel).Then cellswere treated with 30μg/ml ZnONPs and MTS analysis was performed to detect cell viability at 24h after treatment. Data are representative of three independent experiments (n=6 for each group)and values are expressed in mean ± SEM.^**^*p*< 0.01. N.S.=Not significant(Bottom panel). **(c)** FACS analysis of cells stained with LysoSensor Green DND-189. The effect of vehicle orZnONPs(30μg/ml) treatment on acidic milieu was analyzed 24h after treatment. Images are representative of three independent experiments.

**Figure S7 Interplay between damagedmitochondria and ROS generation**

**(a)**FACS analysis of cells stained with 2'7'-Dichlorofluorescin diacetate. The effects of vehicle orZnONPs(30μg/ml) treatment on intracellular ROS were analyzed 24 h after treatment. Images are representative of three independent experiments. **(b)** A549 cells were pretreated with NAC(10mM) followed by 30μg/ml ZnONPs treatment. Western-blot analysis of LAMP2, LAMP1, p62 and LC3B expression levels were performed at 24h after treatment. β-Actin served as loading control. Images are representative of three independent experiments. **(c)** FACS analysis of cells stained with TMRE. The effect of vehicle or ZnONPs(30μg/ml) treatment on mitochondrial membrane potential was analyzed 24h after treatment. Images are representative of three independent experiments.
