# Supplementary material for: Zinc oxide nanoparticles harness autophagy to induce cell death in lung epithelial cells
Source: Cell Death Dis. 2017 Jul 27;8(7):e2954–. doi: 10.1038/cddis.2017.337 (PMC5550878; doi:10.1038/cddis.2017.337)

Figure S1

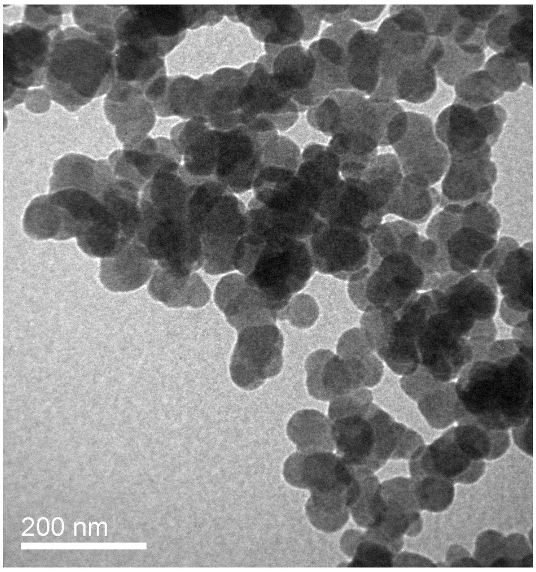

ZnONPs

Figure S2

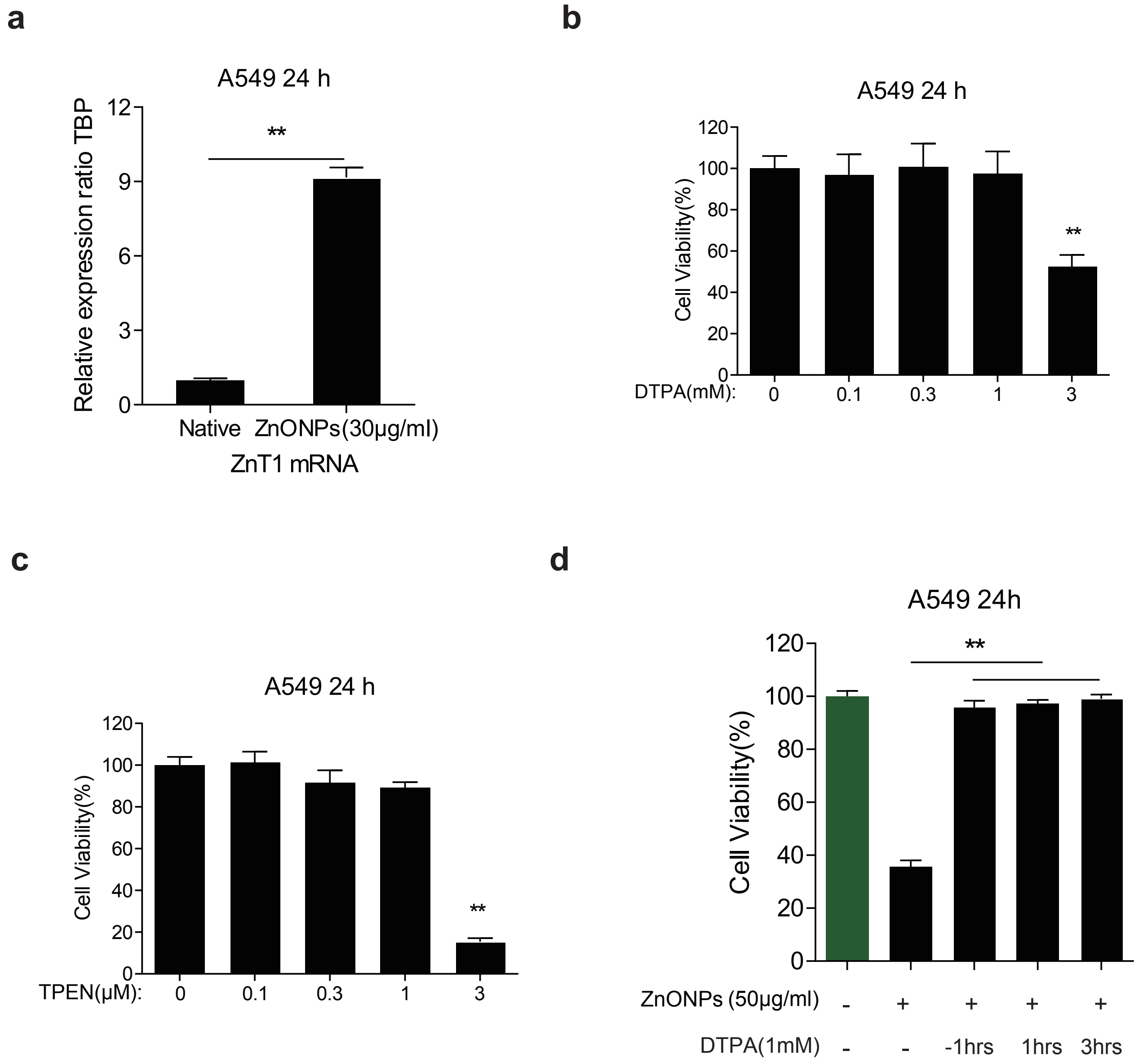
a
A549 24 h
Relative expression ratio TBP
12
9
6
3
0
**
Native
ZnONPs(30μg/ml)
ZnT1 mRNA
b
A549 24 h
Cell Viability(%)
120
100
80
60
40
20
0
**
DTPA(mM): 0 0.1 0.3 1 3
c
A549 24 h
Cell Viability(%)
120
100
80
60
40
20
0
**
TPEN(μM): 0 0.1 0.3 1 3
d
A549 24h
Cell Viability(%)
120
100
80
60
40
20
0
**
ZnONPs (50μg/ml) - + + + +
DTPA(1mM) - - -1hrs 1hrs 3hrs

Figure S3

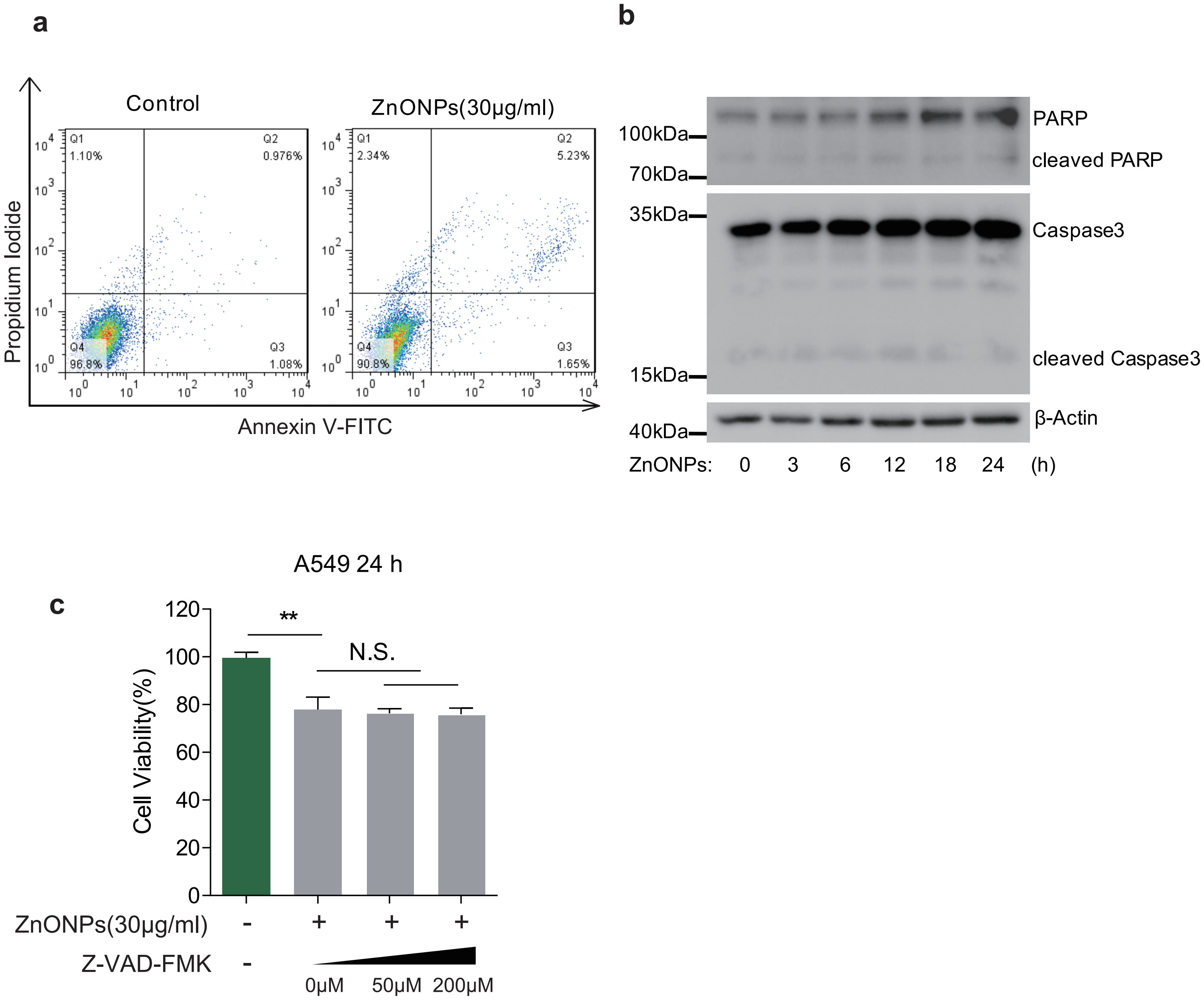

a
Control
ZnONPs(30μg/ml)
Propidium Iodide
Annexin V-FITC
Q1 1.10%
Q2 0.976%
Q4 96.8%
Q3 1.08%
Q1 2.34%
Q2 5.23%
Q4 90.8%
Q3 1.65%
b
100kDa
70kDa
35kDa
15kDa
40kDa
PARP
cleaved PARP
Caspase3
cleaved Caspase3
β-Actin
ZnONPs: 0 3 6 12 18 24 (h)
c
A549 24 h
Cell Viability(%)
**
N.S.
ZnONPs(30μg/ml) - + + +
Z-VAD-FMK -
0μM 50μM 200μM

Figure S4

a

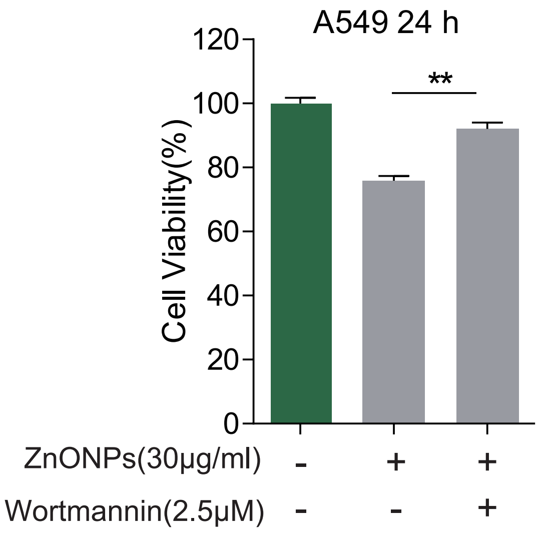

b

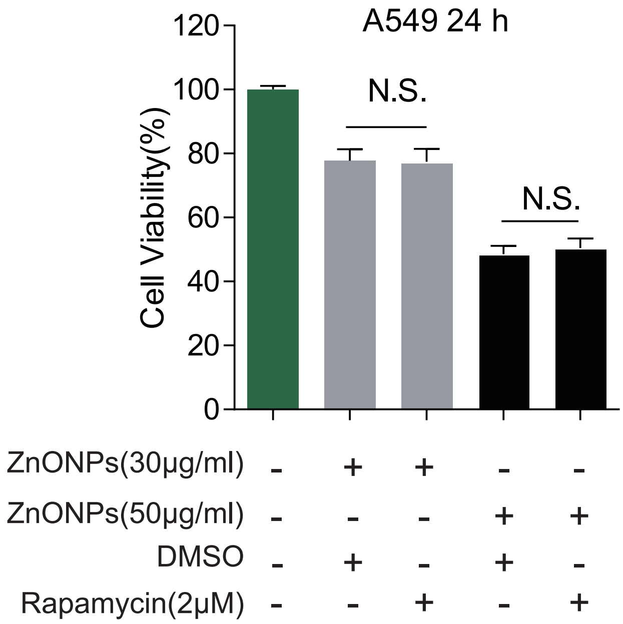

c

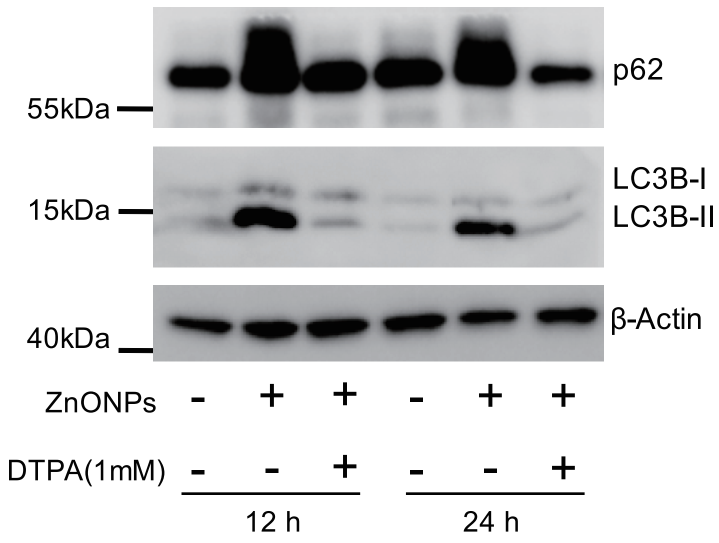

Figure S5

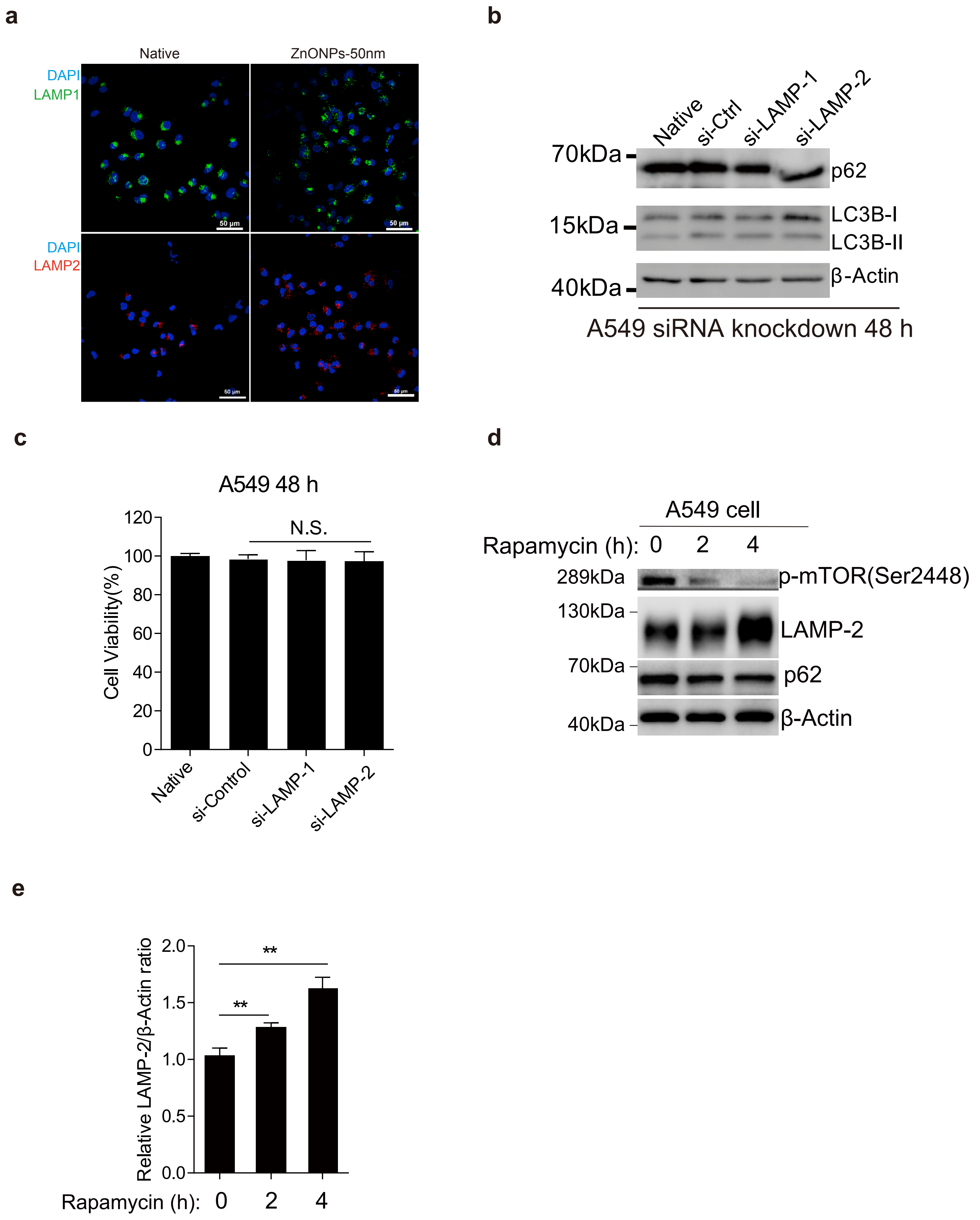
a
Native
ZnONPs-50nm
DAPI
LAMP1
DAPI
LAMP2
50 μm
b
Native
si-Ctrl
si-LAMP-1
si-LAMP-2
70kDa
p62
15kDa
LC3B-I
LC3B-II
40kDa
β-Actin
A549 siRNA knockdown 48 h
c
A549 48 h
N.S.
Cell Viability(%)
120
100
80
60
40
20
0
Native
si-Control
si-LAMP-1
si-LAMP-2
d
A549 cell
Rapamycin (h): 0 2 4
289kDa
p-mTOR(Ser2448)
130kDa
LAMP-2
70kDa
p62
40kDa
β-Actin
e
Relative LAMP-2/β-Actin ratio
2.0
1.5
1.0
0.5
0.0
**
**
Rapamycin (h): 0 2 4

Figure S6

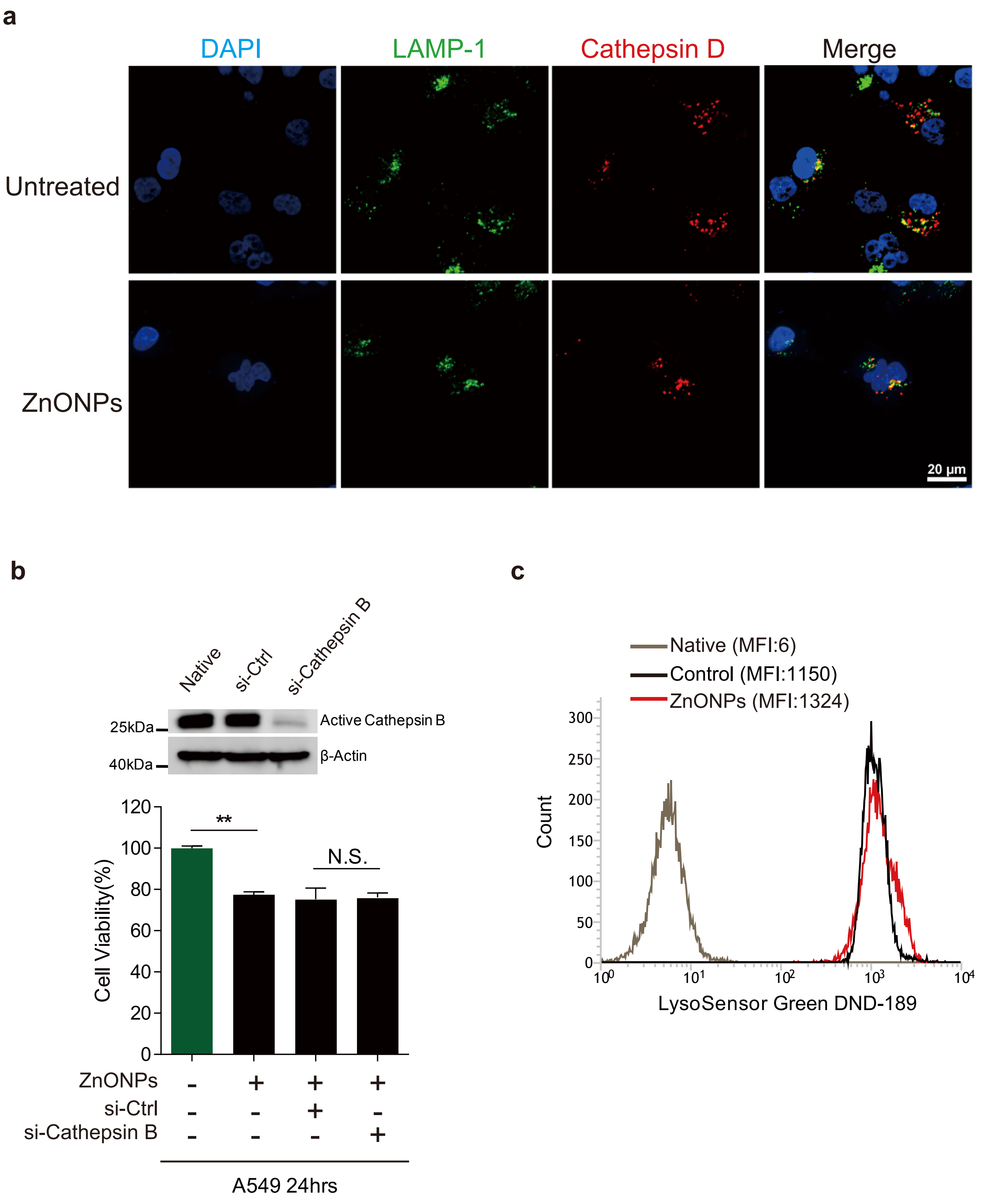
a
DAPI
LAMP-1
Cathepsin D
Merge
Untreated
ZnONPs
20 µm
b
Native
si-Ctrl
si-Cathepsin B
25kDa
40kDa
Active Cathepsin B
β-Actin
Cell Viability(%)
120
100
80
60
40
20
0
**
N.S.
ZnONPs
si-Ctrl
si-Cathepsin B
A549 24hrs
c
Native (MFI:6)
Control (MFI:1150)
ZnONPs (MFI:1324)
Count
300
250
200
150
100
50
0
LysoSensor Green DND-189

Figure S7

a

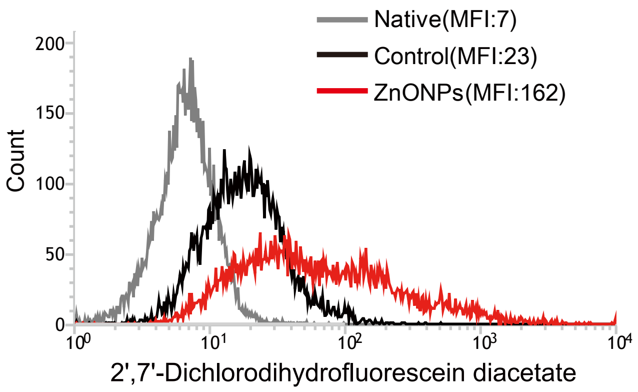

b

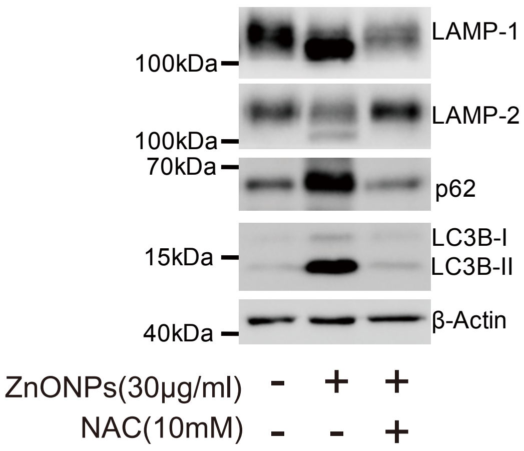

c

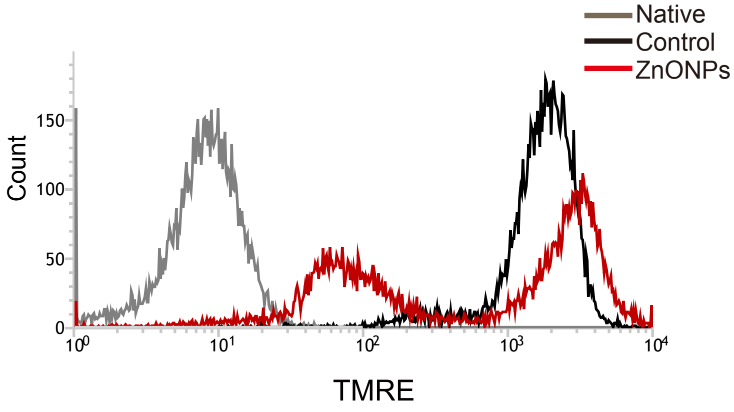

Supplement: Supplementary Figures [file cddis2017337x2.pdf]
